# Time to adjuvant treatment for newly diagnosed pediatric brain tumors in the molecular era: a single center retrospective study

**DOI:** 10.1007/s00381-026-07419-1

**Published:** 2026-08-04

**Authors:** Kenneth L. Virgin, Michael Fletcher, Jinan Ayub, Hannah Harmsen, Laurie L. Ackerman, Jignesh K. Tailor

**Affiliations:** 1 Department of Neurological Surgery, Indiana University School of Medicine, Indianapolis, IN USA; 2 Indiana University School of Medicine, Indianapolis, IN USA; 3 Indiana University Melvin and Bren Simon Comprehensive Cancer Center, Indianapolis, IN USA; 4 Department of Pathology and Laboratory Medicine, Indiana University School of Medicine, Indianapolis, IN USA; 5 Riley Hospital for Children, Indiana University School of Medicine, 705 Riley Hospital Drive, Suite 1601, Indianapolis, IN 46202 USA; 6 Herman B Wells Center for Pediatric Research, Indiana University School of Medicine, IN Indianapolis, USA

**Keywords:** Molecular classification, Treatment delay, Precision oncology, Radiotherapy timing, Targeted therapy, Low-grade glioma

## Abstract

**Purpose:**

Molecular testing has become a cornerstone in pediatric neuro-oncology but relies on time intensive workflows at specialized centers. We hypothesized that time to adjuvant therapy has increased since the incorporation of molecular testing.

**Methods:**

In this retrospective case series, data were collected from Riley Hospital for Children in Indianapolis from the pre-molecular era (2007–2009) and the molecular era (2023–2025). Patients aged ≤ 21 years who received initial surgery for a primary brain tumor were included. The primary outcome of interest was the time from initial surgery to initiation of adjuvant treatment.

**Results:**

A total of 140 patients met inclusion criteria, including 83 who received adjuvant therapy as part of the initial postoperative treatment plan. Time to adjuvant treatment was significantly shorter in the pre-molecular cohort (24.5 vs 34.2 days). Multivariate analysis demonstrated that this prolonged time to treatment was associated with the use of targeted therapies that rely on molecular testing for diagnosis, and increased patient referrals to external institutions during the molecular era. The most cited reasons for delay in adjuvant treatment were final pathology report pending, family decision making, and shunt-related factors.

**Conclusion:**

Time to adjuvant therapy was longer in the molecular era, and this effect was attributable to evolving treatment paradigms, specifically the adoption of targeted therapies related to molecular diagnosis and increased reliance on external treatment centers. These results underscore modifiable, system-level barriers in the delivery of precision oncology which represent key opportunities to improve timeliness of care and, ultimately, patient outcomes.

**Supplementary Information:**

The online version contains supplementary material available at 10.1007/s00381-026-07419-1.

## Introduction

The molecular roots of tumors have been recognized for over a century [1] but the trend towards molecular diagnosis in pediatric CNS tumors hit an inflection point in 2010 when a consensus meeting accepted the existence and clinical relevance of 4 distinct subgroups of medulloblastoma [2]. The 2016 WHO Classification of Tumors of the Central Nervous System [3] broadly incorporated molecular testing and marked the start of the “molecular era” of pediatric neuro-oncology. These molecularly defined subgroups respond differently to treatment and can thus be managed more or less aggressively to maximize efficacy and minimize adverse effects [4, 5 ]. Various tumors have been further classified using molecular markers, allowing precise tumor-specific therapy [6, 7 ]. Most notably, low-grade gliomas with BRAF V600 mutations are effectively managed with MAPK pathway inhibitors [8, 9 ]. Molecular studies have revealed that recurrent tumors are often defined by a molecular signature distinct from the original tumor, thus necessitating novel treatment strategies [10–12]. Large scale adoption of methylation assays is now aiding in difficult to diagnose tumors [13–15]. These molecular tests are refining our understanding of tumor biology and enabling patient-specific treatment.

Although molecular testing has improved outcomes for several tumor types, it is more time-intensive than conventional histological analysis, often resulting in delays between resection and initiation of adjuvant therapy. Lengthy delays in adjuvant therapy have been associated with decreased progression-free survival for medulloblastoma [16] and pilocytic astrocytoma [17]. It is therefore essential that extraneous delays be identified and minimized in oncology. Pediatric Brain tumors are rare with less than a few hundred cases of each subtype in the USA per year [18]. Thus, many institutions, like ours, do not have access to specialized in-house molecular testing and must rely on distant centers for molecular diagnosis. Currently, no guidelines dictate the appropriate time to postpone treatment while awaiting molecular diagnosis nor do they set standard turnaround times for molecular results. We therefore sought to evaluate the impact of incorporating molecular testing on the time to initiation of adjuvant therapy at our institution. We hypothesized that the time to adjuvant treatment had increased in the molecular era. This study was designed as a preliminary, hypothesis-generating analysis to inform future multi-institutional investigations, which are currently underway.

## Methods

This study was approved by the institutional review board at Indiana University and Riley Hospital for Children with a waiver of informed consent.

### Patient population and study design

We conducted a retrospective chart review of patients at Riley Hospital for Children between January 2007 and December 2009 as well as between July 2023 and June 2025. Patients were included if they were ≤ 21 years old with a primary brain tumor and underwent initial neurosurgical intervention for diagnosis or treatment. Patients were excluded if they had undergone prior neurosurgical intervention for tumor diagnosis.

### Data collection

Data were collected from the hospital electronic medical record. The primary outcome of interest was the time from initial surgery to initiation of adjuvant treatment. Secondary outcomes included initial adjuvant treatment employed, whether adjuvant treatment was performed internally or at an external institution, time to initial histological diagnosis, whether slides were sent for second opinion histological diagnosis, time from initial surgery to molecular diagnosis, readmission between surgery and adjuvant treatment, and reason for readmission in such cases (categorized based on Chotai et al. [19]). Time to histological diagnosis was defined as the time to the first signed pathology report containing a tumor diagnosis based on histological evidence and excluding intraoperative reports. Time to molecular diagnosis was defined as the time to receive signed results from the first molecular study.

In 3 cases of the pre-molecular cohort, the start date of adjuvant treatment was not explicitly listed but inferred utilizing the end date of a standardized regimen. In cases where data were unavailable, no other estimates or data imputation were performed, and the specific variable was excluded from analysis.

### Statistical analysis

We reported descriptive statistics for all patient variables, expressing categorical variables as numbers and percentages and continuous variables as mean with standard deviation. For comparative analysis, Wilcoxon rank-sum test was used to assess if distributions differed. For categorical variables, Fisher's exact test was used for 2 × 2 tables, and chi-square tests were used for larger tables. In instances where the expected count for any cell was below 5, Monte Carlo simulation was applied to the chi-square analysis to estimate the *p*-value. A *p*-value < 0.05 was considered significant for this and all analyses. All analyses were performed using RStudio v4.5.1.

Multivariable linear regression was performed to evaluate factors associated with time from surgery to initiation of adjuvant therapy, modeled as a continuous outcome (days). Standardized mean differences (SMDs) were calculated to assess baseline imbalance, with values > 0.1 considered meaningfully different. Given the presence of baseline imbalance between cohorts, as assessed by SMDs, we constructed a parsimonious multivariable model incorporating key clinical variables most likely to influence time to adjuvant therapy, including molecular era, second opinion histological diagnosis, first-line adjuvant therapy, location of treatment, initial surgery, and maximum tumor diameter. Tumor diameter was modeled as a continuous variable scaled per 5 mm to improve interpretability. The effect estimate was reported as beta coefficients with 95% confidence intervals.

Model diagnostics demonstrated no substantial violations of linear regression assumptions. Sensitivity analysis using log-transformed time to treatment yielded similar results with respect to the direction and interpretation of key predictors. Radiation therapy reached statistical significance in the transformed model, without altering the primary conclusions (data not shown).

## Results

We identified 140 patients from Riley Hospital for Children in Indianapolis who met inclusion criteria, including 69 patients from the pre-molecular period (2007–2009) and 71 from the molecular period (2023–2025). Table 1 demonstrates relevant patient characteristics. The pre-molecular and molecular cohorts did not significantly differ in age, sex, ethnicity, area deprivation index (ADI) state decile [20], tumor location, or final pathology of the tumor. Race distribution differed (*p* = 0.030), with a lower proportion of White patients and higher representation of minority groups in the molecular cohort. Private insurance was more common in the pre-molecular cohort (50.7% vs 29.6%), whereas public insurance (60.6% vs 40.6%) and no insurance (8.7% vs 9.9%) were more common in the molecular cohort (*p* = 0.035). The pre-molecular cohort had a smaller mean maximum tumor diameter (40.0 mm vs 48.9 mm; *p* = 0.012) and a lower prevalence of midline shift (6.1% vs 18.3%; *p* = 0.038). The pre-molecular cohort more often underwent resection (82.6% vs 62.0%) than biopsy (17.4% vs 38.0%) as the initial surgery (*p* = 0.008). There was also a trend toward longer time to discharge in the molecular cohort (9.5 vs 18.5 days; *p* = 0.058) although these results did not reach statistical significance.

**Table 1:** Summary statistics of pre-molecular and molecular patients

Variable	Pre-molecular	Molecular	*p* value	SMD
Age	7.3 (5.1)	8.2 (5.4)	0.334	0.16
Sex			0.865	0.03
Male	41 (59.4%)	41 (57.7%)		
Female	28 (40.6%)	30 (42.3%)		
Race			0.030	0.41
White	62 (89.9%)	53 (74.6%)		
Black	6 (8.7%)	14 (19.7%)		
Asian	0 (0.0%)	3 (4.2%)		
American Indian/Alaska Native	0 (0.0%)	2 (2.8%)		
Unknown	1 (1.4%)	2 (2.8%)		
Ethnicity			1.000	0.03
Not Hispanic	61 (88.4%)	62 (87.3%)		
Hispanic	8 (11.6%)	9 (12.7%)		
Insurance			0.035	0.44
Private	35 (50.7%)	21 (29.6%)		
Public	28 (40.6%)	43 (60.6%)		
Uninsured	6 (8.7%)	7 (9.9%)		
Area Deprivation Index State Decile	5.5 (2.9)	6.3 (3.0)	0.085	0.28
Tumor Max Diameter (mm)	40.0 (17.9)	48.9 (21.3)	0.012	0.45
Patient Under Imaging Surveillance	13 (18.8%)	15 (21.1%)	0.834	0.06
Tumor Location			1.000	0.02
Supratentorial	39 (56.5%)	41 (57.7%)		
Infratentorial	30 (43.5%)	30 (42.3%)		
Leptomeningeal Disease	4 (6.1%)	2 (2.8%)	0.428	0.16
Midline Shift	4 (6.1%)	13 (18.3%)	0.038	0.38
Hydrocephalus	38 (56.7%)	35 (49.3%)	0.399	0.15
Initial Surgery			0.008	0.47
Resection	57 (82.6%)	44 (62.0%)		
Biopsy	12 (17.4%)	27 (38.0%)		
Final Pathology			0.453	0.37
Glial	30 (43.5%)	35 (49.3%)		
Embryonal	20 (29.0%)	10 (14.1%)		
Sellar	4 (5.8%)	7 (9.9%)		
Neuronal/Mixed Neuronal-Glial	5 (7.2%)	5 (7.0%)		
Ependymal	4 (5.8%)	6 (8.5%)		
Germ Cell	4 (5.8%)	3 (4.2%)		
Mesenchymal	1 (1.4%)	2 (2.8%)		
Pineal Region	1 (1.4%)	1 (1.4%)		
Peripheral Nerve	0 (0.0%)	1 (1.4%)		
Other	0 (0.0%)	1 (1.4%)		
Intensive Care Unit Admission	52 (85.2%)	66 (93.0%)	0.169	0.25
Additional Procedures During Admission			0.324	0.24
No Additional Procedures	50 (72.5%)	48 (67.6%)		
Shunt Placement	15 (21.7%)	21 (29.6%)		
Resection	2 (2.9%)	6 (8.5%)		
EVD Placement	3 (4.3%)	2 (2.8%)		
Shunt Revision	0 (0.0%)	2 (2.8%)		
Fiducial Placement	1 (1.4%)	0 (0.0%)		
Craniotomy	0 (0.0%)	1 (1.4%)		
Time from Surgery to Discharge	9.5 (11.8)	18.5 (39.5)	0.058	0.31
Discharge Disposition			0.294	0.27
Home	49 (87.5%)	55 (77.5%)		
In-Patient Rehabilitation	7 (12.5%)	15 (21.1%)		
Outside Institution	0 (0.0%)	1 (1.4%)		

*SMD* standardized mean differences, *EVD* external ventricular drain

**Fig. 1:**
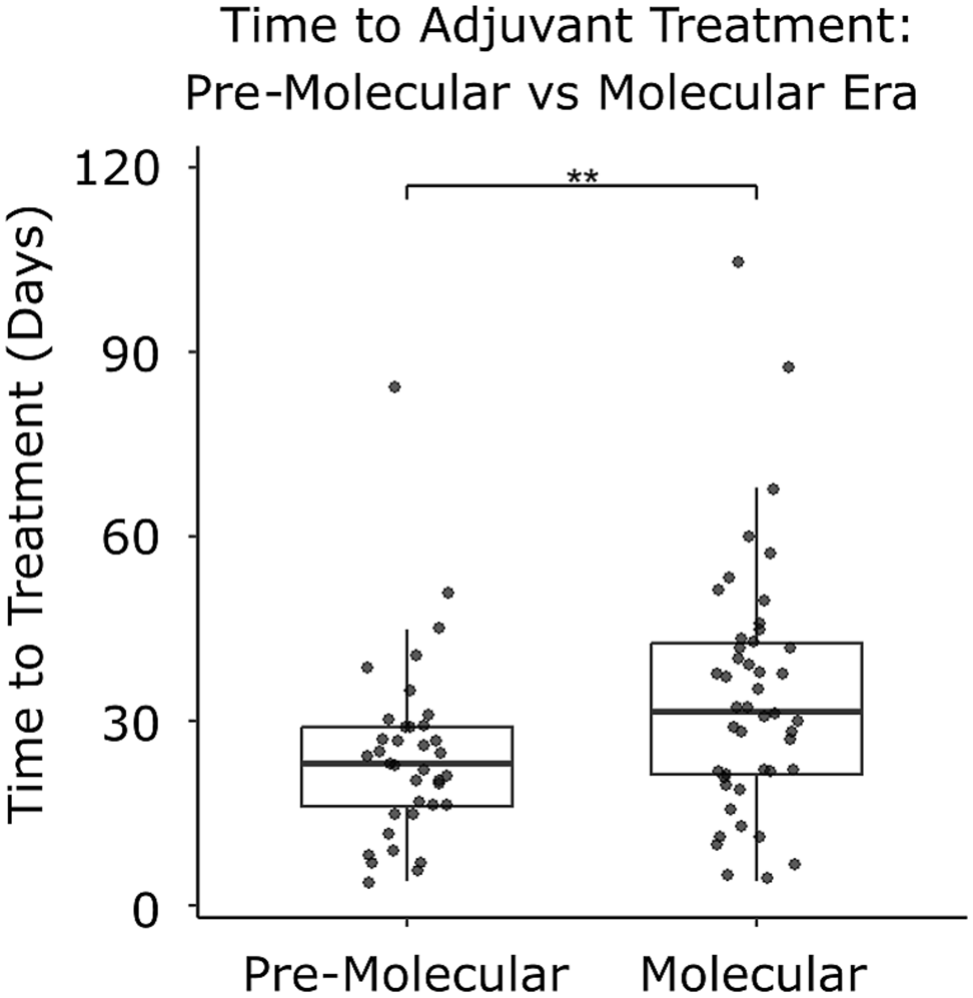
Boxplot of pre-molecular and molecular era time to adjuvant treatment (*n* = 83). Unadjusted analysis shows longer time to treatment in the molecular cohort. The Wilcoxon rank-sum test was used for statistical analysis. **p < 0.01

Our primary outcome of interest, time to adjuvant treatment, was ascertained in 37/69 patients in the pre-molecular and 46/71 patients in the molecular cohort. Patients were excluded from this analysis if they underwent a period of watchful waiting before initiating adjuvant therapy or if the time to treatment could not be ascertained from available chart data. Time to adjuvant treatment differed between cohorts (24.5 vs 34.2 days; *p* = 0.009) as seen in Fig. 1. Multiple secondary outcomes also varied (Table 2). The first-line adjuvant therapy employed in the pre-molecular cohort included radiation (56.4%) and chemotherapy (43.6%) whereas the molecular cohort included radiation (48.9%), chemotherapy (25.5%), and targeted therapy (25.5%). Despite longer time to adjuvant therapy, there was no significant difference in the rate of readmission between surgery and adjuvant therapy. Across all patients, readmissions were due to shunt-related events (6), further tumor debulking (4), wound dehiscence (1), and emesis (1). One patient died before the planned adjuvant treatment could be administered in the molecular cohort.

**Table 2:** Primary and secondary outcomes of pre-molecular and molecular patients

Variable	Pre-molecular	Molecular	*p* value	SMD
Time from Surgery to Adjuvant Treatment	24.5 (14.8)	34.2 (20.2)	0.009	0.54
First Line Adjuvant Therapy			0.002	0.83
Radiation	22 (56.4%)	23 (48.9%)		
Chemotherapy	17 (43.6%)	12 (25.5%)		
Targeted	0 (0.0%)	12 (25.5%)		
Type of Radiation			0.644	0.25
Photon	14 (63.6%)	13 (56.5%)		
Proton	6 (27.3%)	9 (39.1%)		
Unknown	2 (9.1%)	1 (4.3%)		
Location of Adjuvant Therapy			0.010	0.61
In-State Institution	38 (97.4%)	36 (78.3%)		
Out-of-State Institution	1 (2.6%)	10 (21.7%)		
Time to Histological Diagnosis	4.0 (3.7)	4.2 (2.1)	0.035	0.08
Second Opinion Sought for Histological Diagnosis	5 (7.2%)	17 (23.9%)	0.010	0.47
Molecular Studies Performed	0 (0.0%)	60 (84.5%)	N/A	N/A
Time from Surgery to Molecular Results	N/A	26.2 (12.4)	N/A	N/A
Time from Histological Diagnosis to Molecular Results	N/A	23.8 (12.6)	N/A	N/A
Readmission Between Surgery and Adjuvant Treatment	3 (8.6%)	8 (17.0%)	0.338	0.25
Reason for Readmission			0.258	1.41
Shunt-Related	2 (66.7%)	4 (50.0%)		
Tumor Debulking	0 (0.0%)	4 (50.0%)		
Wound Dehiscence	1 (33.3%)	0 (0.0%)		
Emesis	0 (0.0%)	1 (12.5%)		

*SMD* standardized mean differences

**Fig. 2:**
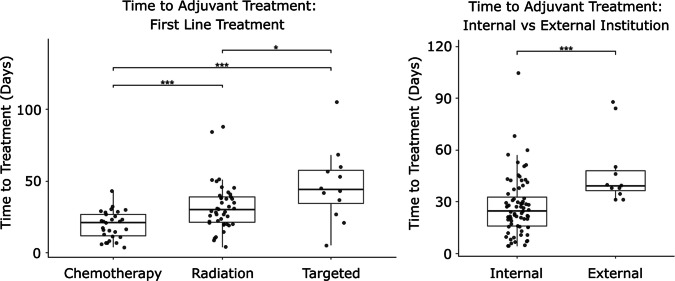
Boxplots of key factors related to time to adjuvant treatment in unadjusted and potentially confounded analysis (*n* = 83). Time to treatment was greatest for targeted treatment and least for chemotherapy when all patients were pooled together (*n* = 83). Similarly, the time from surgery to adjuvant treatment was greater when patients were referred to an external institution for treatment. The Wilcoxon rank-sum test was used for statistical analysis. *p < 0.05, ***p < 0.001

To further probe the differences in time to treatment between cohorts, we pooled all patients who underwent adjuvant treatment (*n* = 83) to perform exploratory univariate analysis and found that chemotherapy was associated with a shorter time to adjuvant treatment compared to radiation (19.3 vs 32.2 days; *p* < 0.001) and targeted therapy (19.3 vs 46.9 days; *p* < 0.001) as seen in Fig. 2. Radiation was also associated with a shorter time to adjuvant treatment than targeted therapy (32.2 vs 46.9 days; *p* = 0.022). Additionally, adjuvant therapy at an external institution was associated with a longer time to treatment (47.3 vs 27.2 days; *p* < 0.001). In addition to these exploratory analyses, multiple variables demonstrated meaningful imbalance between cohorts (SMD > 0.1), consistent with evolving clinical practice and patient populations over time.

**Fig. 3:**
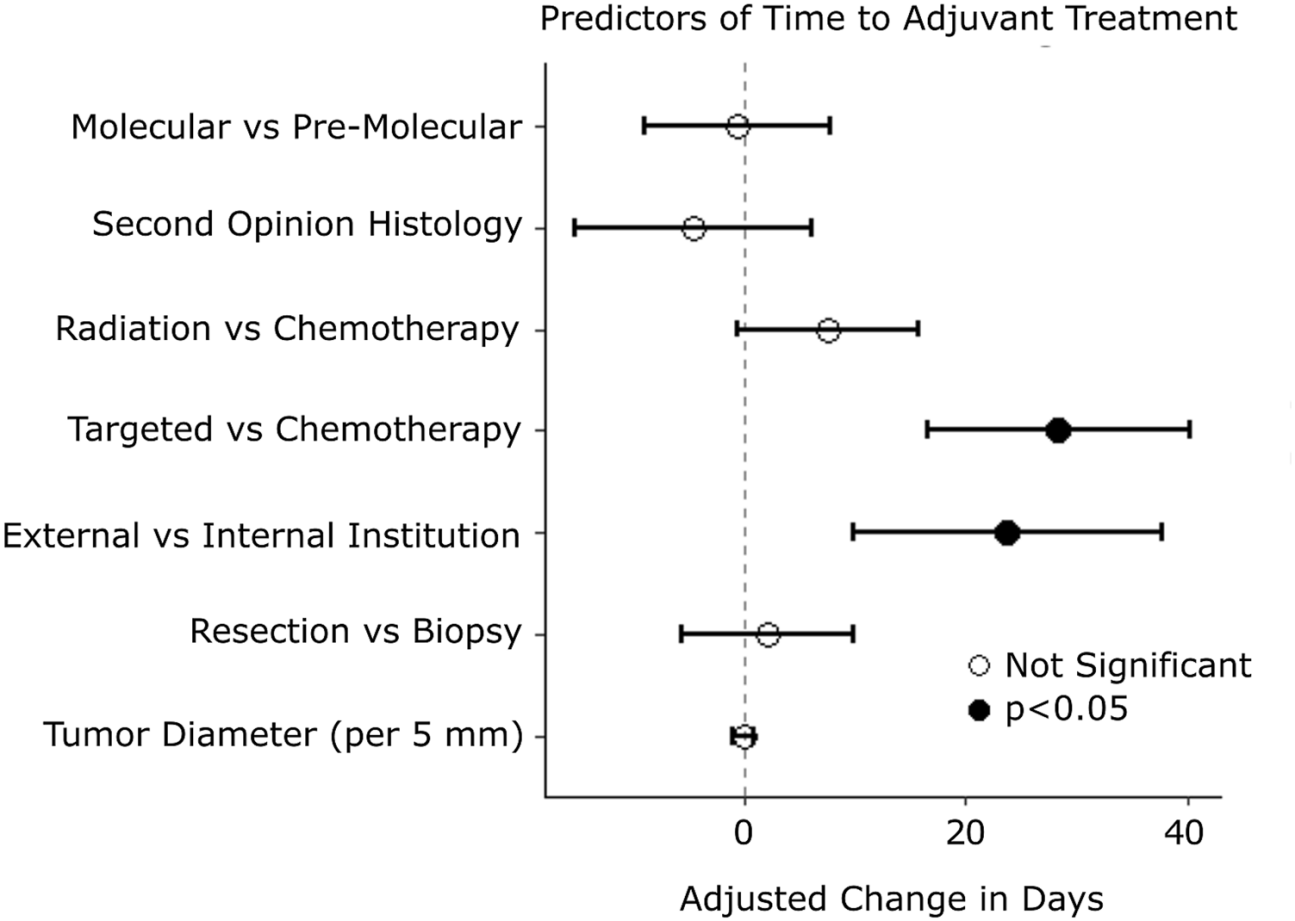
Forest plot of multivariate analysis of predictors of time to adjuvant treatment. Targeted therapy compared to chemotherapy and treatment at an external institution were associated with longer times to adjuvant treatment whereas treatment era was not. Radiation compared to chemotherapy trended toward longer times but did not reach statistical significance

We thus performed a multivariate analysis to find the effects of molecular era, second opinion histological diagnosis, first-line adjuvant therapy, location of treatment (internal vs external institutions), initial surgery (biopsy vs resection), and maximum tumor diameter on time to adjuvant therapy. As seen in Fig. 3 and Supplemental Table 1, targeted therapy and external treatment were independently associated with significantly longer time to adjuvant treatment. No significant associations were observed for other treatment modalities, pre-molecular vs molecular status, initial surgery type, or tumor diameter. While radiation therapy was associated with a longer time to treatment, this did not reach statistical significance. Sensitivity analyses in which additional variables with SMD > 0.1 were individually added to the model did not affect directionality of the effect estimates but statistical significance varied, with external treatment becoming non-significant in one model and radiation therapy becoming significant in multiple models. Targeted treatment demonstrated significance across all models.

The time to initial histological diagnosis was higher in the molecular cohort by a narrow margin (4.0 vs 4.2 days; *p* = 0.035), and more cases were sent to an outside institution to obtain a second opinion histological diagnosis (7.2% vs 23.9%; *p* = 0.010). Molecular testing was completed in 84.5% of patients in the molecular cohort and the time between surgery and the molecular diagnosis was 26.2 days, significantly longer than traditional histological diagnosis (*p* < 0.001). Molecular tests included targeted DNA sequencing, gene fusion testing, copy number alteration analysis, RNA expression profiling, DNA methylation profiling, chromosomal microarray, and germline genetic testing.

As part of our review, we noted any factors that were linked to a delay in treatment in one or more clinical notes. We performed this review for the purpose of identifying potential areas of improvement and did not compare the results of the two cohorts as this would be confounded by differences in note writing styles. The cited reasons for delay were grouped into 5 major categories: social factors, systematic delays, patient complications, health insurance related, and further surgical intervention (see Table 3). The 3 most cited causes of delay were final pathology report pending, family decision making, and shunt-related factors. Neither shunting rates nor time to shunt placement postoperatively were significantly different between cohorts and shunt-related factors contributed to delays in both cohorts.

**Table 3:** Barriers to adjuvant therapy initiation specifically mentioned in patient charts

Category	Barrier to adjuvant therapy
Social factors	Family decision-making
	Transportation difficulties
	Language barrier
	Miscommunication with family
Systematic delays	Final pathology report pending
	Second opinion pathology pending
	Transfer of care to external institution
	Delays in Imaging
	Clinical trial enrollment
Patient complications	Shunt-related
	Gastrostomy tube placement
	Pulmonary complications
	Patient rehabilitation
	Unstable neurologic status
	Urinary tract infection
	Fever
	Emesis
	Cerebral salt wasting
	Diabetes insipidus
	Acute kidney injury
	Wound dehiscence
Health insurance related	Insurance authorization
	Lack of insurance
Further surgical intervention	Second look surgery
	Fiducial placement

## Discussion

Recent advancements in molecular testing have provided opportunities for accurate diagnosis and precise risk stratification, allowing treatment plans to be tailored to the individual’s risk profile [21–23]. Molecular testing has become widely adopted into pediatric neuro-oncology and was performed in 84.5% of patients at our institution during the molecular era. Our results suggest that the molecular era is associated with prolonged time to treatment which may offset some positive outcomes of refined diagnosis.

Prolonged time between surgery and initiation of adjuvant therapy has been associated with poor outcomes in pediatric brain tumors. Delayed diagnosis and treatment are associated with decreased progression free survival in medulloblastoma [24, 25 ] and pilocytic astrocytoma [17]. Studies have also shown that recurrent tumors may develop molecular signatures distinct from their primary tumors thus necessitating additional molecular testing for effective management [10]. Moreover, recurrent tumors can be more biologically aggressive due to the influence of inflammatory changes and wounding [26, 27 ]. It stands to reason that decreasing the time to adjuvant therapy should be a primary goal of management. In this study, four patients required further debulking procedures, and one patient passed away before adjuvant therapy could be administered. These events emphasize the need for earlier recognition and diagnosis of intracranial lesions and earlier adjuvant treatment after surgery.

In our multivariate analysis, we revealed that prolonged time to treatment was associated with modifiable system-based barriers, such as use of targeted therapies that rely on molecular testing, and patient referral to external institutions. Targeted therapy is inherently linked to molecular diagnosis, which took an average of 26.2 days to return and was significantly longer than the approximately 4 days for histological diagnosis. In previous studies, the implementation of molecular testing has been shown to lead to delays in refined diagnoses [28]. At our institution, these delays are compounded by reliance on specialized external centers, many of which employ proprietary techniques that are not readily reproducible locally. The rarity of pediatric CNS tumors [18] may also reduce cost-effectiveness at local centers.

As part of a similar systematic shift, the reliance on external institutions at our institution has increased in the molecular era. From 2004 to 2014, Indiana had a proton therapy center associated with Indiana University, but our patients now rely on out-of-state centers for this and other forms of treatment. Patients receiving treatment at an out-of-state institution experienced a 20-day delay in treatment overall, emphasizing disparities that may exist for patients without internal treatment centers.

The systems producing prolonged time to treatment have the potential to produce inequities in healthcare access. In low- and middle-income countries, access to testing is limited, which may contribute to worse outcomes for children with brain tumors [29]. The scarce availability of in-house molecular pathology contributes to the diagnostic uncertainty faced by these populations, as well as in rural populations, who experience a 15% higher mortality risk from brain cancer than their urban counterparts [29]. Further research is needed to determine whether disadvantaged populations are disproportionately affected by the systematic shifts that define the molecular era and what workflow modifications can decrease treatment disparities.

Many differences were present between the pre-molecular and molecular era beyond adjuvant treatment patterns. Our results indicate that more tissue is being sent for second opinion diagnosis, indicating increased collaboration between institutions. Patients are also more likely to undergo biopsy as the initial surgical management in the molecular era. Pediatric brain biopsies such as brainstem biopsies have become more common, allowing accurate, integrated diagnosis with reduced adverse effects compared to resections [30]. Biopsies may yield limited sample and risk inconclusive results and longer turnaround times. Despite this risk, our multivariate analysis did not demonstrate a longer time to adjuvant treatment in biopsies compared to resections.

Although not statistically significant, we noticed a substantial upward trend in time to discharge in the molecular cohort. Our data do not correspond with overall trends in pediatric hospitalizations during this period and may thus be disease or location specific [31]. Interestingly, tumor diameter tended to be larger in the molecular cohort, along with higher rates of midline shift. This could be related to the molecular cohort being defined by a population more frequently reliant on public insurance [32] or it could be related to other factors [33] which might delay diagnosis and lead to increased tumor size and mass effect at presentation. However, our study was not designed for an analysis of these variables; maximum tumor diameter is not an ideal measurement for tumor volume, and midline shift was determined by the radiologist report and not manual assessment and should thus be interpreted with caution.

In Table 3, we identify cited causes of delays in adjuvant therapy beyond molecular diagnosis in our patients. These causes were based on the documentation of the treating physicians and are thus subjective but informative. Many are related to surgical and shunt complications inherent to invasive procedures and provide further support for research to improve shunt reliability and surgical safety. Others relate to systemic problems such as the time to integrated pathological diagnosis and clinical trial enrollment. Communication barriers and delays caused by family decision making may be addressed by early and repeated discussions on therapy options to address the cognitive and emotional aspects of decision making [34].

### Limitations

Several limitations exist in this study. Our two cohorts were not matched and reflected large differences in patient population and management patterns over the 15-year gap between cohorts. Additionally, some associations in the multivariate analysis were sensitive to model specification in sensitivity analyses, likely reflecting the limited sample size and correlation between clinical variables. The retrospective single institution analysis introduces selection bias and limits generalizability. These results may be least reproducible at larger institutions where molecular testing and adjuvant treatments are performed in-house.

### Future directions

At our institution, we are implementing changes to our diagnostic workflow to reduce the time to molecular diagnosis for our patients. Considering the high rate of molecular testing, we are working to send tissue for molecular testing on post operative day 1 instead of waiting for a histological diagnosis to initiate the process. Future development and application of intraoperative testing using mass spectrometry [35–37], ramen spectroscopy [38], or other technologies may also accelerate diagnosis.

Our findings reflect the experience of a single institution and highlight institution-specific barriers to timely molecular diagnosis and initiation of adjuvant therapy. To better define national trends and inform future guidelines, we are collaborating with the Society for Pediatric Neurosurgical Oncology (SPNO) and the AANS/CNS Section on Pediatric Neurological Surgery on a multi-institutional cross-sectional study evaluating molecular diagnostic workflows and adjuvant treatment patterns in pediatric brain tumor patients. This effort may facilitate comparison of institutional practices and support standardized approaches to molecular diagnosis and care delivery.

## Conclusions

In this single-institution retrospective study, unadjusted analysis suggested longer time to adjuvant therapy in the molecular era. Multivariable modeling demonstrated that this delay was driven by increased use of targeted therapies and care delivered across external institutions. These findings highlight modifiable, system-level and treatment-specific factors as key barriers to timely adjuvant therapy. A larger, national study is therefore needed to validate these findings across diverse practice settings, quantify the relative contribution of care fragmentation and evolving treatment paradigms, and identify actionable targets to standardize care pathways and improve timeliness of therapy delivery.

## Supplementary Information

Below is the link to the electronic supplementary material.

ESM 1 (DOCX 19.3 KB)

## Data Availability

No datasets were generated or analysed during the current study.
