# Supplementary material for: Time to adjuvant treatment for newly diagnosed pediatric brain tumors in the molecular era: a single center retrospective study
Source: Childs Nerv Syst. 2026 Aug 4;42(1):322. doi: 10.1007/s00381-026-07419-1 (PMC13433393; doi:10.1007/s00381-026-07419-1)
Supplement: Supplementary file 1 [file 381_2026_7419_MOESM1_ESM.docx]

Supplementary Table 1. Multivariable linear regression of time to adjuvant treatment

Outcome: time from surgery to adjuvant treatment (days)

|  | Estimate (Days) | 95% Confidence Interval | p-value |
| --- | --- | --- | --- |
| Molecular vs Pre-Molecular | -0.54 | -8.84 to 7.76 | 0.897 |
| Second Opinion Histology | -4.54 | -15.18 to 6.11 | 0.398 |
| Radiation vs Chemotherapy | 7.57 | -0.58 to 15.72 | 0.068 |
| Targeted vs Chemotherapy | 28.36 | 16.49 to 40.24 | <0.001 |
| External vs Internal | 23.76 | 9.84 to 37.68 | 0.001 |
| Resection vs Biopsy | 2.11 | -5.60 to 9.82 | 0.586 |
| Tumor Diameter (per 5 mm) | 0.04 | -0.88 to 0.95 | 0.935 |
